# Exploring Female EFL Teachers’ Professional Agency for Their Sustainable Career Development in China: A Self-Discrepancy Theory Perspective

**DOI:** 10.3389/fpsyg.2022.906727

**Published:** 2022-06-23

**Authors:** Xiaolei Ruan, Auli Toom

**Affiliations:** ^1^School of Foreign Studies, Anhui University, Hefei, China; ^2^Centre for University Teaching and Learning (HYPE), Faculty of Educational Sciences, University of Helsinki, Helsinki, Finland

**Keywords:** professional agency, self-discrepancy, female EFL teachers, metaphor, China

## Abstract

A large and growing body of literature has investigated the role of teachers’ agency in their career trajectories. However, far too little attention has been paid to English as a Foreign Language (EFL) teachers’, especially female EFL teachers’, professional agency for their career development in the Chinese higher education setting. To address this gap, this study explores female EFL teachers’ professional agency from a self-discrepancy theory perspective, namely, how the participating teachers have perceived discrepancies in their professional development and how they have enacted their professional agency to realize sustainable development. Based on a metaphor investigation of 167 teachers and interviews with nine of them, the current study found that (1) there are certain discrepancies between female EFL teachers’ self-guides and actual selves concerning their professional identity construction; (2) female EFL teachers’ professional agency is manifested in the continuum of iteration, practical evaluation, and projectivity processes, as well as in the entity of personal and environmental factors; and 3) female EFL teachers’ professional agency and gender identity are closely intertwined with each other. This study can offer implications for teacher agency research and female teachers’ sustainable development at large.

## Introduction

The sociocultural perspective in educational research (Johnson, 2009) has generated a large body of literature focusing on teachers’ professional development, wellbeing, and life experience (Kayi-Aydar, 2019), and it is claimed that more studies should be devoted to listening to and understanding teachers’ voice in China (Wen and Zhang, 2017). Female English as a Foreign Language (EFL) teachers have constituted a majority of the teaching cohort in the higher education setting in China, who are labeled with multiple identities, such as gender, subject/discipline, and profession. There are certain impediments to female EFL teachers’ sustainable development (Jiang, 2012). Teacher agency can motivate teachers as “reflective practitioners” to actively engage themselves in making decisions and exerting influences on their pedagogical choices and their career development at large (Toom et al., 2015; Ruan and Zheng, 2019). This study aims to explore the perceived discrepancies of female EFL teachers and the dynamic processes of how they have enacted their professional agency to realize sustainable career development.

## Literature Review

### Teacher Agency for Sustainable Career Development

To be an agent is to intentionally make things happen by one’s actions, and agency embodies the endowments, beliefs, self-regulatory capabilities, and distributed structures through which personal influence is exercised (Bandura, 2001; Archer, 2002). *The chordal triad of human agency* proposed by Emirbayer and Mische (1998) has provided illuminating account for the understanding of agency, which epitomizes *iteration*, *practical evaluation*, and *projectivity*, referring, respectively, to the selective reactivation by agents of past patterns of thought and action, agents’ capacity to make practical and normative judgments among alternative possible trajectories of action, in response to the emerging and changing situations, as well as the imaginative generation by agents of possible future trajectories of action, when they creatively reconfigure their thoughts and actions according to their hopes, desires, and sometimes, even fears for the future.

Teacher agency is considered as teachers’ essential professional capability, which means that teachers as learners and agents can intentionally plan, act or not to act, and reflect upon their behavior and, hence, develop teachers’ competencies and exert influence upon their career trajectories (Toom et al., 2015, 2021). On the basis of Emirbayer and Mische’s model, Priestley et al. (2015) proposed an ecological framework for teacher agency, which is relational and contextual and is, thus, socially embedded in the continuum of “past–present–future” and is developed in the dynamic integration of personal situation, school context, and sociocultural factors. In other words, it brings a perspective of teacher agency into discussion and allows one to perceive teacher agency as relational, contextual, and socially embedded (Toom et al., 2021). As such, teachers are able to fulfill multiple meaning-making efforts to fulfill their goals and planning in the classroom and in the professional community (van der Heijden et al., 2015).

In recent climate, researchers in China have conducted a large number of empirical studies to explore the efficacy aspects of teacher agency. Zhang (2017) analyzed the environmental factors and personal factors serving as mechanism for teacher agency. Qi et al. (2020) investigated the *status quo* of teacher agency for professional development and its influencing factors. Tao and Gu (2016) pinpointed that “selectivity” and “compensation” are two major modes when teachers enact their agency. Gao et al. (2018) found that decision-making and acting are two striking features of teacher agency. Li and De Costa (2019) revealed that EFL teachers’ enactment of agency was a result of negotiation with contextual constraints and resources. Ruan (2020) revealed that agency beliefs, agency practice, and agency inclination have penetrated teachers’ agency enactment. Wang and Wang (2020) argued that negative emotions, for example, sense of oppression, is an important factor to activate agency and make a difference. The empirical studies listed before adopted various qualitative and quantitative methods, such as interview, observation, and questionnaire, to address the complexities of teacher agency in the Chinese setting, which has provided implications for an in-depth understanding of the features and influencing factors of teacher agency. However, what motivate teachers to exercise their agency and how do they exercise their agency? How agency is enacted in various professional tasks, such as teaching, research, and teacher learning? How agency is found within a certain group of tertiary teachers, to be more specific, female EFL teachers? Can other data collection methods be used to enhance and enrich our understanding of the issue? These questions are crucially important and await to be addressed.

### Female EFL Teachers’ Career Development in China

Female teachers constitute a large cohort of the whole teaching population in the higher education context in China. According to the official statistics from the Ministry of Education [ME] (2021), there are altogether 1,345,940 female teachers in Chinese higher education institutions, yet there are far less female teachers with senior professional titles in universities than their male counterparts. (Among those who hold a full professor title, there are 31.7% female teachers, 18.4% supervisors of doctoral programs, and 37.4% master program teachers.) The role conflicts of female teachers are manifested in the relationship between the family and career, and the relationship between teaching and research (Zhou, 2016).

As for female EFL teachers, although their population dominates the whole teaching cohort, but there are often bottlenecks restricting their professional development (Jiang, 2012). According to Meng and Chen (2015), there are certain divisions of beliefs concerning family roles and social identities between male teachers and female teachers, and the research engagement among male EFL teachers is found to be far more active than their female counterparts. There are certain reasons contributing to the aforementioned phenomenon, for instance, the glass ceiling effect (Morrison et al., 1987), suggesting women encounter invisible barriers that prevent them from rising to the upper rungs of career ladder (Hymowitz and Schellhardt, 1986), the loss of agency (Smith, 2011), etc.

Agency is found to be closely related to female identity, and female teachers’ professional agency is intertwined with their gender identity (Lasky, 2005). According to the post-structuralist assumption, gender identity is something individuals do or actively perform, rather than some qualities they have (Davies, 1997). The dimensions of “selecting” and “controlling” can help women make decision, construct identity, and shape their life course (Evans and Biasin, 2017). According to Rudolph et al. (2018), female EFL teachers negotiate their lived experiences with positionality, and they utilize their agency to trouble essentialized discourses of identity in their personal–professional lives. Therefore, this study aims to explore female EFL teachers’ professional agency for their sustainable career development.

### A Self-Discrepancy Theory Perspective on Teacher Agency

According to Higgins (1987), there are three basic domains of the self: the ideal self, the ought self, and the actual self, referring separately to the attributes to possess ideally, attributes one should possess, and attributes one actually possess. The ideal self-domain and the ought self-domain, namely, the valued self-end states are defined as “self-guides” (Van Hook and Higgins, 1988).

Moreover, Higgins argued that it is not enough to distinguish among different domains of self. It is also necessary to discriminate among self-state representations by considering whose perspective on the self is involved and, hence, the standpoints on the self. Combining each of the domains of the self with each of the standpoints on self-yields different types of self-representations.

Self-discrepancy is the gap between two of these self-representations. The self-discrepancy theory postulates that these different representations of the self can be contradictory to each other, which inevitably results in certain degrees of emotional discomfort. The theory claims that people are motivated to reach a condition where our self-concept matches our personally relevant self-guides (Higgins, 1987, p.321).

Furthermore, Higgins (1987) cogently argues that the congruity between the different selves can produce positive emotions, thus strengthening and enhancing motivations, while clashes or conflicts between different selves might induce negative emotions with detrimental impacts on motivations. It is noteworthy that Higgins (1987) also pinpointed that the self-discrepancy theory may have implications in “predicting positive emotions” and “initiating and directing action,” as well as “moderating motivational and emotional consequences” (p. 336).

The notion of self-discrepancy is closely related to the construct of teacher agency. As mentioned earlier, “selecting,” “acting,” and “compensating” are the major dimensions and features found in teacher agency. As Gao and Xu (2014, p. 153) postulated that “the discrepancies among teachers’ representations of ‘actual,’ ‘ought,’, and ‘ideal’ selves are likely to become the driving forces in their professional development and career pursuit.” By employing self-regulatory strategies and seeking external help, teachers can “reduce the degree of self-discrepancy and regain their motivations” (Yuan et al., 2016, p. 221). That is to say, the exercise of teacher agency forges the processes of teachers’ problem-solving competencies and their deliberation in tackling obstacles to bridge the gap between the professional ideals and the realities (Ruan et al., 2020). Therefore, this study assumes that the self-discrepancy theory can serve as a theoretical lens in understanding female EFL teachers’ agency enactment and sustainable development. In line with this, the following research questions are addressed:

(1) Which discrepancies have the participating teachers perceived in their career development?

(2) How have they enacted their professional agency to bridge the gap of different self-guides?

## Methodology

Qualitative research is interpretative and the inquirer is typically involved in a sustained and intensive experience with participants (Creswell, 2013), which suggests that a qualitative design addresses people’s “lived experience” and, thus, is “fundamentally well-suited for locating the meanings people place on the events, processes, and structures of their lives” (Huberman and Miles, 2002). The current study seeks to explore female EFL teachers’ perceived discrepancies and agency enactment; thus, the qualitative approach fits the “interpretive” nature of the study and is about to realize “thick description” by dealing with “whys” and “hows” and provide insights into social phenomenon dynamics.

To be concrete, a phenomenological qualitative research design was adopted in the current study, in which an initial metaphor investigation with 167 female EFL teachers in China informed in-depth interviews with nine of them during the data collection, not only generating an overall picture of the group but also zooming in on individual cases.

### Participants

Through a comprehensive consideration of purposive sampling, convenience sampling, and snowball sampling (Patton, 2002), the study selected 167 female EFL teachers at the university level in China to take part in the first-round metaphor investigation: (1) We selected participants with more than 5 years of teaching experiences, who are supposed to have relatively stabilized and consolidated understanding of their profession (Huberman, 1989); (2) We attempted to recruit teachers of varied background, such as region and type of the university, teachers’ professional title, courses they teach, and their teaching length, based on the principle of maximum variations; (3) We further enlarged the possible research population by utilizing our social network.

After careful reading of the metaphor investigation, we solicited representative answers which have the potential to generate more findings in the subsequent interviews and asked for the willingness and availability of the participants for the follow-up data collection. Subsequently, nine teachers were recruited in the second-round interview.

The basic information of the participants are presented, respectively, in Tables 1, 2.

**TABLE 1:** Teachers taking part in the first-round metaphor investigation (*N* = 167).

Region		University type		Professional title		Teaching length		Educational background		Courses to teach	
Eastern China	45%	Project 985 university^a^	5%	Full professor	8%	5–10	42%	Doctoral degree	11%	English major	54%
Central China	38%	Project 211 university^b^	47%	Associate professor	31%	11–15	29%	Master’s degree	83%	College English	46%
Western China	17%	Other universities	48%	Lecturer	59%	16–20	16%	Bachelor’s degree	6%		
				Other	2%	> 20	13%				

*^a^Project 211 is the Chinese government’s new endeavor aimed at strengthening about 100 universities and key disciplinary areas as a national priority for the twenty-first century.*

*^b^Project 985 is a constructive project for founding world-class universities in the twenty-first century.*

**TABLE 2:** Teachers taking part in the second-round interview (*N* = 9).

Pseudonym	University type	Professional title	Teaching length	Educational background	Course to teach
Rose	A provincial^a^ normal university	Full professor	21	Doctoral degree	English major
Carnation	A foreign studies university	Lecturer	18	Doctoral degree	College English
Sunflower	A science and technology-oriented Project 985 university	Associate professor	12	Doctoral student	College English
Tulip	A comprehensive type Project 211 university	Lecturer	6	Master’s degree	English major
Orchid	A comprehensive type Project 211 university	Associate professor	8	Master’s degree	English major
Mimosa	A foreign studies university	Associate professor	5	Doctoral degree	English major
Lily	A finance-oriented provincial university	Lecturer	11	Master’s degree	College English
Violet	A provincial normal university	Lecturer	15	Master’s degree	English major
Osmanthus	A provincial medical university	Lecturer	6	Doctoral student	College English

*^a^A provincial university in China means a university governed by a province.*

### Data Collection

#### Metaphors

Metaphor is a powerful artifact and a tool for opening up possible conceptual territories for exploration of their connections and dynamics in constructing knowledge (Yob, 2003). According to Seferoğlu et al. (2009), metaphors are windows into how human beings conceptualise the world and the reality. A metaphor is considered as schema projected into another schema, through which meanings are expressed (Levin and Wagner, 2006). It is not only a linguistic phenomenon and a rhetoric device but also a way of thinking and cognition (Lakoff and Johnson, 2003), which plays an important role in how humans define and understand the world (Moghadam and Samar, 2020). In the field of teacher research, metaphors here can be used to explore teachers’ emotions and to interpret the sociocultural contexts (Gosselin and Meixner, 2015). This study intends to identify the possible discrepancies of EFL female teachers for their professional development; a metaphor fits this purpose by allowing a nuanced description and understanding of their self-guides and actual selves. In this study, we asked the participating teachers to use metaphors to express their identity commitment (for instance, *What kind of metaphor would you use to express your self-guide?* and *what kind of metaphor would you use to express your actual self?*^1^). Through a process of a pilot study, revising instructions, and formal investigation, 167 valid metaphor questionnaires were collected and analyzed.

#### Semi-Structured Interviews

We thematically analyzed the metaphor questionnaire and selected nine of the 167 teachers to attend the second-round data collection—semi-structured interviews (Kvale, 2007). Due to the consideration of availability and convenience, many of the interviews were conducted online, especially for those participants who were geographically far away. There were three rounds of interviews: (1) the first-round interview focusing on the participants’ learning and professional histories; (2) the second-round interview concerning the milestone events in the participants’ career development processes; and (3) the third-round interview exploring the major factors influencing teachers’ agency enactment. Each interview lasted about 0.5–1 h. By assuring the participants their answers would be only for research purpose, and their real names and their schools would never appear in public, we recorded the interviews.

### Data Analysis

First, we conducted a frequency count of the metaphor questionnaires collected to get a general scenario of female EFL teachers’ identity commitment; second, we conducted a qualitative content analysis of the participants’ description of how and why they had selected each metaphor; third, we transcribed the interview recordings verbatim, which generates 276,213 words for further analysis; finally, we conducted thematic analysis by coding, categorizing, synthesizing, and theorizing (Saldaña, 2009) the material: (1) importing the texts into the computer-assisted qualitative data analysis (CAQDA) means named MAXQDA (version Analytics Pro 2018) and immersing in the data by repeated readings; (2) coding the text initially and continuing to add, delete, and revise existed codes, shaping them into code systems and sub-menus under different classes; (3) getting the text printed to conduct manual coding and comparing the results of it with the software coding; (4) seeking the suggestions from experts and peers regarding the inconsistencies between software coding and manual coding; and (5) generating 98 items after the open coding process (Table 3).

**TABLE 3:** Protocol of the coding process.

Theory	Theme	Category	Codes
Self-discrepancies perceived	Self-guides	Overall	Being refined both externally and internally; multi-talented; efficient; charming; elegant; Jack of all trades; motivated
		Teaching	Imparting knowledge; shaping students’ character; gardener; candle; beacon light; like teachers and like friends; quality education; students-centered; organizer of classroom activities; communicating; interacting; teaching students in accordance of their aptitude
		Research	Integrating teaching with researching; researcher; teacher researcher; productive; grant application; famous scholar; research quality; research team
		Teacher learning	Live and learn; innovation; stay current; content knowledge; pedagogical knowledge; technological knowledge
	Actual self	Overall	Busy and occupied; tiresome; hard to balance; confused; role conflicts; self-doubts; lack of understanding; accept and love myself
		Teaching	Discipline status; language as tools; humanistic literacy; settling down and making a living; mechanical repetition
		Research	Bottleneck; pragmatic; competitive; reading literature; taking part in academic meetings; searching for a direction; publish or perish; pressure for title promotion
		Teacher learning	Seminar; workshop; lectures; academic conferences; teaching contests; visiting study home and abroad
Professional agency enacted	Time	Iterative	Learning experience; working history; research experience; habit; style; attitude; principle; measures
		Practical-evaluative	Analyzing; planning; evaluating; selecting; execution; task-management; self-control; self-supervision; time management; fragment of time; self-reflection; external help
		Projective	Short-term career planning; long-term career planning
	Context	Personal	Knowledge; attitude; character; emotion; responsibility; professional ethics; teaching methods
		Environmental	Classroom; students; colleagues; leaders; family members; friends; institution culture; school policy; social expectation

With an aim to ensure the trustworthiness of the research (Huberman and Miles, 2002), the following attempts have been made: (1) a multitude of sources of data were collected to realize triangulation; (2) the results of data analysis were given back to the participants for member check; and (3) the comparison between manual coding and software coding was conducted to increase reliability.

## Findings

The following section presents the research findings based on the two research questions proposed.

### Self-Discrepancies Perceived by Female English as a Foreign Language Teachers

To address the first research question, the following two sections present the findings both globally and concretely on the basis of metaphor investigation and interview.

#### Inevitable Conflicts? Self-Discrepancies Perceived by Multiple Roles

Through an analysis of the metaphor investigation, it is found that the participating teachers tend to use human beings, plants, animals, natural phenomenon, and sports events to express their identity commitment (Table 4). When referring to self-guides, female teachers applied different types of person, such as ‘‘sculptor,’’ ‘‘gardener,’’ ‘‘ferryman,’’ and ‘‘engineer,’’ to demonstrate that teachers intend to guide their students and teachers are full of creativity and execution; metaphors of ‘‘tree root,’’ ‘‘pine tree,’’ ‘‘wintersweet,’’^2^ ‘‘sunflower,’’ and ‘‘broken butterfly cocoon’’ suggest that the qualities valued by the female teachers are resilience, optimism, and self-growth; metaphors of ‘‘rainbow,’’ ‘‘kaleidoscope,’’ ‘‘candles,’’ and ‘‘breeze’’ expressed teachers’ expectation of varied roles, their dedication, and longing for a refined temperament. When they refer to their actual selves, metaphors of ‘‘explorer’’ and ‘‘running man’’ suggested teachers’ continuing attempts for self-improvement; metaphors of ‘‘octopus,’’ ‘‘dandelion,’’ ‘‘dancing leaves in the wind,’’ and ‘‘migrating birds’’ demonstrate that female teachers endeavor to seek direction against external hardships; and metaphors of ‘‘winding mountain road,’’ ‘‘seesaw,’’ ‘‘panacea,’’ and ‘‘Alipay’’^3^ reveal the participants’ determination to realize diversity in the developmental paths.

**TABLE 4:** Examples of the metaphors provided by the female EFL teachers.

Self-guides			Actual self		
	
People (37%)	Animals and plants (41%)	Others (22%)	People (12%)	Animals and plants (37%)	Others (51%)
Sculptor, gardener, painter, dancer, tourist guide, engineer, ferryman, etc.	Tree root, pine tree, wintersweet, spring silkworm^a^, rose, broken butterfly cocoon, sunflower, etc.	Sun, umbrella, breeze, triathlon, rainbow, kaleidoscope, candle, compass, etc.	Explorer, running man, etc.,	Cat, gecko, octopus, worker bee, dancing leaves in the wind; dandelion, migrating birds, etc.	Winding mountain road, seesaw, spinning top, panacea, Alipay, rock, balance scale, a cup of tea, coffee, footsteps in sandbeach, etc.

*^a^In China, people often compare teachers to silkworm and candle to eulogize their dedication spirit, with its origins from the ancient poem about love “Till the end of life a silk worm keeps spining silk; Till burning itself out a candle goes on lighting us” written by the poet Li Shangyin of late Tang Dynasty.*

#### Concrete Manifestations: Teaching, Research, and Teacher Learning

Interview with the teachers suggests that self-discrepancies perceived by female EFL teachers are manifested in the harmonious coexistence in rhetoric and the role conflicts of different identity commitments in reality. As Orchid from a Project 211 university said:

**Extract 1.** *Female EFL teachers should not only do teaching and research well, but also try to become life-long learners. What’s more, we have the obligation to look after the family. These tasks are hard enough to tackle with, which requires us to become “iron women.”*

It can be found from the quote that female EFL teachers’ perceived role conflicts and self-discrepancies are manifested in multiple professional tasks, namely, teaching, research, and teacher learning commitments.

### Self-Discrepancies in Teaching

Research findings show that the English curriculum reform is one of the biggest challenge teachers face in their teaching. Many teachers expressed their determination to “keep pace with the changing requirement of teaching,” while many realized “hardships and challenges” in the curricular reforms.

Carnation, with 18 years of teaching experience in a renowned foreign studies university, showed her earnest love for teaching as a middle-aged veteran teacher:

**Extract 2.** *I think no matter what courses to teach, it is a process of familiarization and enhancement. I am not that type of teacher who always stay in the comfort zone, say, if the textbook remains the same, the teacher could use the same teaching plan for several rounds.*

Mimosa earned her doctoral degree from a world-renowned university abroad. Although she’s eager to implement reforms in her classroom, she faced many challenges.

**Extract 3.** *I really wanted to flip the classroom by assigning lots of homework (searching for a topic, attending online courses) before and after the class while encouraging my students to hold discussions and interact with each other in class. When I saw the score they have given to me in the teaching evaluation session, I was so disappointed. Perhaps I was too strict with them.*

### Self-Discrepancies in Research

Teachers also referred to their contradictions and embarrassment in their research engagement. Tulip from a comprehensive university expressed her helplessness regarding the requirements made by her university for the promotion of professional titles:

**Extract 4.** *Although the university distinguishes teachers as “research-oriented” and “teaching-oriented” in the promotion processes, generally speaking, the requirements about research are strict. How many journal articles have you published? Where have you published these articles? These are all quantified. It is pleased to see teaching has been given more weight in the promotion processes recently, but you can never quantify teaching like research.*

Violet mentioned that “the combination of teaching and research” advocated by the university is almost a castle in the air:

**Extract 5.** *My research direction is theoretical linguistics, which, honestly speaking, has nothing to do with my teaching. If you want to stay up-dated in research, you should read, read, and read, therefore you can figure out what the research hot spots are. I don’t believe in the nexus between research and teaching.*

### Self-Discrepancies in Teacher Learning

Female EFL teachers consider themselves as “lifelong learners,” yet the actual role conflicts made them less concentrated on workplace learning and self-improvement. Osmanthus had been a college English teacher at a medical university before she pursued her doctoral university in another university.

**Extract 6.** *As a full-time teacher with more than ten periods of classes to teach each week, I need to prepare for the lesson, arrange classroom activities, coach students, and attend many meetings. It is almost impossible for me to study. Therefore, I decided to quit my job and become a full-time doctoral student. Only in this way can I concentrate on my own research.*

Rose mentioned that both the summer vacation and winter vacation are good opportunities to learn because she can stay at home or in the library to get herself “charged.”

**Extract 7.** *Before the summer vacation and winter vacation start, I usually borrow some books from the library to read, which are closely related to my research. Another thing which is also very important is “going out”. During my 20 years of teaching career, I have applied several times of visiting study abroad. Being they several weeks or one year in length, they are really beneficial to me.*

### Professional Agency Enacted by Female English as a Foreign Language Teachers

It is found that teachers enact their professional agency actively to bridge the gap between their self-guides and actual selves, which is manifested in the continuum of history, present, and future and is situated in the dynamic interaction between the self and environment.

#### Being the Shapers of Their Own Career Trajectories: The Time Dimension of Agency

The enactment of teacher agency is closely related to teachers’ learning experience, professional history, their belief system, and their behavior style. Lily, a lecturer from a finance-oriented provincial university, contributed her motivation for continuing professional development to her self-control developed when she was a student.

**Extract 8.** *Every time I go back home, I am surround by my daughter. After she goes to bed, it is usually mid-night. I am afraid that I should fall sleep with my her, so I set an alarm clock to wake me up and then go to the study room for lesson preparing or literature reading. This habit originates from my high school days, when both of my parents were too busy to supervise my study. So I am kind of an independent and a clear-visioned person.*

The female EFL teachers in this study also emphasized the significance of selecting, acting, and reflecting. They claimed that one should not only have the self-efficacy to believe that she can do it but also have the ability to transfer her plan into reality. Sunflower recounted her experience in teaching the academic English writing course.

**Extract 9.** *Academic writing, to our knowledge, is a bit dull and daunting course if I taught students about the thesis structure, academic words and phrases. I intentionally borrowed an idea from a Korean reality TV show called The Running Man to let my students “learning by doing”. They went to the library in groups to search relevant literature, send pictures to the online discussion group, and went back to the classroom for group discussion. In this way, their critical thinking, team-work, and problem solving skills are crafted.*

Both short-term and long-term planning determine the direction where agency will development. Osmanthus believed that in order to balance family and career, it is crucial to have plans and act them out.

**Extract 10.** *Family and career are two important lines which needs meticulous planning. In different stages of life, there are different emphases. When the two lines intersects at some point, self-management counts. When you accompany the kid and coach his or her homework, you’d better forget about your research and teaching identities and when you immerse yourself in your work, you should not consider too much about your family. Anyhow, you can never do your work well without a loving and supporting family.*

Violet referred to a “to-do list” when she talked about self-management and self-supervision.

**Extract 11.** *When I was a student, I usually create lots of “to-do-lists”. It gives me sense of fulfillment when I finished the items on the lists. Now summer vacation is coming, I made a to-do-list for myself again.*

#### Singing the Duet of Individual and Environment: The Context Dimension of Agency

The dynamic interaction between the self and environment has penetrated the process of teacher agency enactment. For one thing, how teachers exercise their agency is deeply influenced by their personal attitude and ability.

Carnation assumed that if the teacher wants to “offer students a drop of water,” he or she should “have at least a bowl of water.” Additionally, “this bowl of water” needs constant refreshing.

**Extract 12.** *Our millennial students are really talented and smart with good English proficiency. Therefore, I do believe in the value of “information gap” between one student and the other, and between the students and the teacher. Keeping learning as a habitual thing is really important.*

Sunflower believed that teaching is the ongoing processes of attempting, learning, and reflecting.

**Extract 13.** *This semester, I began to teach students English newspaper reading course, which is a completely new realm for me. I kept “teaching by learning” and after a whole round of teaching has completed, I realized there are many limitations. It doesn’t matter, I will take my time to grow.*

For another, it is found in this study that environmental factors also shape how teachers enact their agency. Mimosa referred to herself as a motivated and ambitious teacher, yet her husband “not recognizing her work” frustrated her from time to time.

**Extract 14.** *Once I talked to him (my husband) that my ultimate goal is to be promoted as a full professor. He replied me without a second thought: “How come you dream to be a full professor!” As a researcher with science background, he can hardly understand the value of humanistic research. He believes that most of my work done are less meaningful. Therefore, he hopes me not do any “extra work” except for ten periods of classes each week.*

Rose contributed her growth to her female role models, with her mother and grandmother exerting positive influences.

**Extract 15.** *Both my grandmother and mother often said: “I like to see things grow”. This simple but powerful sentence has left me with great and lifelong impact.*

Lily realized that institutional culture and institutional support are very important for female EFL teachers’ sustainable development.

**Extract 16.** *My university is one with industrial features, which leaves very limited space for female College English teachers. We do not have much discourse power and the path for promotion is really narrow. I often have the feeling of going into a dead end.*

Orchid emphasized a positive and healthy ecology for female teachers’ career development.

**Extract 17.** *Our teaching department established a learning community recently, which, I think is a good thing for those with similar research interests to share and discuss. We meet each other every Wednesday afternoon.*

Most of the teachers also showed their expectation for the understanding from the whole society.

**Extract 18.** *We really hope there will be more voices from the society to support us. Family management needs the joint efforts of both the husband and the wife. Those female teachers who have pursuits in their career should not be labeled as irresponsible and selfish.*

The aforementioned sections have addressed the two research questions, respectively, with the metaphor investigation showcasing female EFL teachers’ overall expectations, actual situation, and existing gaps, and the interview demonstrating female EFL teachers’ perceived discrepancies in teaching, research, and teacher learning and complicating how these teachers enact their agency to deal with the discrepancies and role conflicts through the constant endeavors of past, present, and future, as well as the meticulous negotiation with personal, interpersonal, and sociocultural factors.

## Discussion

With an aim to explore female EFL teachers’ perceived discrepancies in teaching, research, and teacher learning and the ongoing dynamics of their agency enactment, this study claims that although there are discrepancies between female EFL teachers’ self-guides and actual selves, teacher agency is a significant entity by which they can bridge the gap between rhetoric and reality and to seek sustainable career trajectories.

### Perceived Discrepancies Regarding Female Teachers’ Professional Development

First, female teachers do have positive self-guides concerning their identity commitment and professional development, while at the same time, they realize that actual selves are restricted by certain internal and external forces. Exploring female teachers’ ideal self, ought self, and actual self through a way of metaphors reveals teachers’ identity construction and negotiation. Metaphors can not only dig deep into teachers’ inner self but it can also reveal the influences of the sociocultural contexts upon teachers’ personal development. This has shown metaphor’s feature of cognition, experience, and dependence on society and culture as a research approach (Harder, 2010). Empirical studies from the perspective of the sociocultural theory have transferred the research focus from language and thought to the complex relationship between the context and discourse use, identity construction, and social ideology (Duffy, 2014).

Second, the self-discrepancies facing female EFL teachers are manifested in the idealized coexistence of different roles and the actual conflicts of these roles in reality. Teachers perceive discrepancies in their professional development from different aspects, such as teaching, research, and teacher learning activities (Tao and Gao, 2017; Ruan et al., 2020). The discrepancies perceived in teaching activities are manifested in the relatively fixed language teaching mode and the ever-changing requirements from curricular reforms, and in the fatigue of teaching based on years of repeated labor and the urgent need for cultivating teaching efficacy; the discrepancies perceived in research are manifested in the existing job promotion structure and teachers’ lack of research literacy, and in the ideal state to combine teaching and research and the actual paralleled and isolated state of the two; the discrepancies perceived in teacher learning are manifested in the ongoing needs for study and the restrictions from different aspects, for instance, teaching commitment and family burden.

Third, when teachers perceive discrepancies, sense of negative emotions emerge, such as lack of motivation and lack of desire for professional development, accompanied with self-doubts, occupational burnout, sense of depression, and sense of frustration, etc. However, it is also the perceived discrepancies and negative emotions which can push teachers change the *status quo*, bridge the gap, mediate the motivation, and carry out meaning-making activities. This, to a great extent, has echoed with Higgins’ (1987) postulation. Teachers, by enacting professional agency, can narrow the gap between their self-guides and actual selves and, hence, realize continuing career development.

### Dynamic Processes of Female Teachers’ Agency Enactment

For one thing, teachers manifested features and qualities of “selecting,” “acting,” “planning,” and “reflecting” when exercising their agency (Tao and Gu, 2016; Gao et al., 2018), and teachers exercised their agency in a dynamic and complicated way development process (Emirbayer and Mische, 1998). Teachers have established a relatively fixed system of beliefs and knowledge based on years of learning, teaching, and professional development, which are the prerequisite and iterative process of teacher agency; teachers attempt to improve classroom teaching, engage in academic research, and conduct workplace learning through intentional selecting, controlling, reflecting, and meaning-making efforts, which are the practical evaluation and ways of realization; teachers have both short- and long-term goals for their career development, and through working out blueprints for their personal development and creating concrete to-do lists, they are able to make themselves more target-driven and more adept in managing behavior, which are the projective and ongoing motivation for future development. To this end, female EFL teachers’ professional agency is manifested in the continuum of history, present, and future. Therefore, *time* is the first important dimension of agency enactment.

For the other, teacher agency is the integration and interaction of personal, institutional, and sociocultural factors (Priestley et al., 2015). As proposed by Chu et al. (2021), factors in the macro-, meso-, and micro-subsytems produce synergetic effects on teachers’ professional development. Teachers’ personal beliefs and cognition toward female’s roles, professional development, teaching, research, and teaching and learning are the important prerequisites for their agency enactment; teacher knowledge, for instance, their content knowledge, pedagogical knowledge, and technological knowledge, are the important pathway for them to exercise agency for learning them to exercise agency for learning; teachers’ positive attitudes and personality regarding professional development are the medicating forces to turn agency beliefs into agency practice. Teachers live neither in isolation nor in vacuum, whose career development is influenced by the situated and social contexts, namely, significant others, classroom environment, institutional culture, and social atmosphere. To this end, *context* is the second important dimension of agency enactment.

### Distinct Features of Female EFL Teachers’ Professional Agency

It is also claimed in this study that female EFL teachers’ professional agency is intertwined with their female identity commitment, which reveals that professional agency is an open entity influenced by gender agency (Lasky, 2005; Rasmussen, 2009). It is found that female EFL teachers face role conflicts between career development and family commitment when they exercise their agency. Female EFL teachers seek work–life balance and attempt to approach their ideal selves and ought selves through continuous performativity (Archer, 2002; Guest, 2002; Cinamon and Rich, 2005). This study further demonstrates the openness and dynamics of identity construction (Billett, 2006), when different identity commitments and roles are interwoven within an individual. Female EFL teachers’ professional agency and professional identity are easily influenced by female’s gender identity.

Based on the aforementioned illustrations, this study claims that teachers’ self-guides determine the projective aspect in the “chordal triad of agency”; their actual self-echoes with the practical evaluative aspect of agency; teachers’ ideal self, ought self, and actual self are influenced by the learning and teaching history in the iterative aspect of agency. For one thing, self-discrepancies are the driving force for teachers’ career development (Gao and Xu, 2014); self-discrepancies help produce motivation (Dörnyei, 2009), which encourages teachers exercise agency to seek self-regulation and external help and to narrow the gap between different self domains; the negative emotions caused by the perceived discrepancies can push teachers regain motivation and carry out meaning-making efforts in a way of exercising agency and turning negative emotions to positive ones (Hökkä et al., 2017). For another, teacher agency is the product of personal and environmental interactions, which is mediated in the sociocultural context with complexity and dynamics. In certain context of time and space, both restrictions and affordance coexist. Personal differences (e.g., motivation, emotion, identity commitment, and experience) and environmental factors have shaped teachers’ personal choice to “take action” or not (Xu and Long, 2020).

## Conclusion and Implications

Female EFL teachers in the higher education setting face inevitable discrepancies between self-guides and actual selves. Through a series of meaning-making efforts in acting and selecting, they enact their professional agency and attempt to narrow the existing gaps and, hence, seek sustainable career development. Based on the current study, it is suggested that (1) female EFL teachers are to establish grand professional ideals; hold self-efficacy in handling complicated relationship between family and career, working and learning, and teaching and research; cultivate positive professional emotions; enhance teacher knowledge (Technological, Pedagogical, and Content Knowledge) and research literacy to promote teaching competencies and teacher leadership; craft the ability in time management, self-control, rational judgment, positive planning, and reflective learning; and seek external help and positive values from role models when enacting their agency; (2) faculties are to encourage teachers to build learning communities, provide a transparent and fair pathway for promotion and continuing study, and offer positive emotional wellness counseling while necessary; and (3) the family members of female EFL teachers and the whole society at large are to eliminate prejudices, give more understanding concerning female teachers’ career development, and show respect for their personal choices and work engagement.

This study adds to our knowledge of female EFL teachers’ agency enactment and offers referential implications regarding female EFL teachers’ professional development; however, it inevitably has its limitations; for example, this study only focuses on the participants’ articulated beliefs and practices through metaphor investigation and interview, yet how these teachers actually do remains vague. Future studies can incorporate research approaches, such as observation and netnography, to reach a more comprehensive understanding of the issue.

## Data Availability Statement

The raw data supporting the conclusions of this article will be made available by the authors, without undue reservation.

## Ethics Statement

Ethical review and approval was not required for the study on human participants in accordance with the local legislation and institutional requirements. The patients/participants provided their written informed consent to participate in this study. Written informed consent was obtained from the individual(s) for the publication of any potentially identifiable images or data included in this article.

## Author Contributions

XR: conceptualization, methodology, funding acquisition, formal analysis, writing – original draft, review and editing, and investigation. AT: conceptualization and writing – review and editing. Both authors contributed to the article and approved the submitted version.

## Conflict of Interest

The authors declare that the research was conducted in the absence of any commercial or financial relationships that could be construed as a potential conflict of interest.

## Publisher’s Note

All claims expressed in this article are solely those of the authors and do not necessarily represent those of their affiliated organizations, or those of the publisher, the editors and the reviewers. Any product that may be evaluated in this article, or claim that may be made by its manufacturer, is not guaranteed or endorsed by the publisher.

## Footnotes

1 In the pilot study, we asked the participants to refer to 3 metaphors identifying their ideal self, ought self, and actual self, yet most of the participants found difficult to distinguish the terms of “ideal self” and “ought self.” Therefore, in the formal data collection session, we only distinguished two types of self-domain, namely, self-guides (ideal self and ought self) and actual self.

2 Wintersweet is a flower which often reaches its full blossom in the freezing cold winter. The spirit of wintersweet has been valued by Chinese people since ancient times to now, which are considered the symbols of resilience and persistence.

3 Alipay is a digital payment in China which claims to remove barriers between different aspects of life so that customers can enjoy a streamlined way of living, empowered by technology. The teacher uses this metaphor to show her ambition to become powerful in different aspects of life.
